# Odontological Society of Pennsylvania

**Published:** 1893-06

**Authors:** 


### ODONTOLOGICAL SOCIETY OF PENNSYLVANIA.

The regular meeting was held at 1228 Walnut Street, Philadelphia, April 8, 1893. After disposing of the routine matters of the evening, the Committee on Clinics, by Dr. A. W. Deane, made a report as follows:

Dr. Genese operated this afternoon on a first bicuspid on the right side. It had received no treatment, and the gum was partly grown over the side of the root. He mounted the crown, soldered the pins, and placed the case in the mouth very satisfactorily.

(The patient was brought in and the work examined by the members present.)

Dr. Deane also stated that Mr. Kerr, representing the Downey Furnace Company, had brought in a portable furnace and baked a piece of porcelain in five to eight minutes, there being no odor or smell.

Dr. Genese, by numerous samples of teeth and crowns passed around to the members, and by drawings on the black-board and oral description, described his manner of using them and their construction. He stated that they could be procured from any dental depot, and they are manufactured by the Wilmington Dental Company.

Mr. Kerr, representing the Downey Furnace Company, said,—

"I am here to answer any questions you may like to ask in regard to its work. The crown is made of any of the teeth that are in use to-day. Grinding the teeth, except for length, is unnecessary. It is not absolutely required that it should fit the root or band. You can use it even if it is too short."

Mr. Kerr described the method of using the furnace, and stated that the time consumed in fusing the crown was only about two minutes.

At this point Dr. Genese read a paper entitled, "On Crowns."

(For Dr. Genese's paper, see page 413.)

After reading the paper, in answer to some questions put by the members, Dr. Genese remarked,—

"I can record about four hundred cases where these have been inserted. In the earlier stages we had some trouble, but by the process of heating the platina all that has been avoided. The only difficulty I had in the earlier operations was that of the platina box pulling out.

"The porcelain is of the very densest character, similar to that of Ash's porcelain, of London, and the more it is baked the better it gets, and the more it shrinks the tighter it is set in the box. There is no fear of its expanding or cracking after it is set. As a crucial test to which it was put I would refer to that of placing it on the anvil before it became cool, and cooling it rapidly within five minutes after soldering. So if there was any possibility of its cracking it would crack then.

"In the mouth, in masticating, they last exceedingly well. You can grind them to the thinnest possible edge without breaking. The pivot fits into the cavity its whole length, and in cementing, the extra cement will enter the box of the tooth and make it a solid crown and the tooth a homogeneous whole. One, two, or three pivots can be placed in the tooth as well as a single one."

In response to a question as to bridgework, the doctor said, "I have a special form for bridgework; not of that kind of crown, but one very similar to it. It has also the platina barbs inserted on the same plan."

Dr. Bonwill followed Dr. Genese, remarking,—

"I would like to congratulate everybody in making a move in the right direction and doing something that has not been done before.

"I see but one thing new about this, and that is the little porcelain strip fastened in the base of the tooth for soldering, which I cannot see is of any use, because a porcelain crown can be made of a shape that it is almost impossible to break in the mouth, if it is made of proper material, and without soldering and without platina; besides, I don't believe in the principle of placing a pin in the crown of the tooth. Many roots, in order to get in a pin of proper size, have to be cut so thoroughly to pieces that they spread and split, and it will be found that the oxyphosphate will be eaten out.

"The Logan crown is this exactly, except that it is infinitely stronger, cheaper, and easier put in. I would not take the trouble to adjust two pins and solder them, when I can do it more readily with all-porcelain English crowns. The only thing about it is a platina lining, which I always cut out to make the room larger in order to make it adjustable. An English crown placed in and thoroughly anchored in the root of the tooth is one of the strongest crowns that has ever been made. Don't use any with a pin in it. Anchor the pin thoroughly with anything of the character of oxyphosphate, and you can put them on without any trouble."

*Dr. Genese.*—My friend Dr. Bonwill has been contradicting himself. He first tells us he does not want a pin in the root, and then tells us to use a strong one. I came here with the idea of a system of pivoting porcelain teeth without reaming them out.

In answer to one objection of Dr. Bonwill's, Dr. Genese said, "My assistant happened to put a silver pin in the tooth I used today, and I thought it was platina. The silver melted in the box, and that left me with one root or one pivot standing in the tooth; to go through the work again and fix the pivot was more labor than I cared to take, so I simply cemented my second pivot into the tooth, as Dr. Bonwill advises, and then I put my tooth on one root and soldered the tooth to one pivot and the other pivot cemented in the root with oxyphosphate in the box of the tooth itself, thus carrying out both ideas, with the exception that I did not ream the roots out for fear of running through the sides and producing irritation.

"I differ with Dr. Bonwill as to the holding of an all-porcelain crown by itself on to the root, either by screwing it into its place or by oxyphosphate, with any porcelain we have at our command at the present time. If you dovetail your root, it must either be a true dovetail or the cavity must run right through the tooth and dovetail it on the grinding surface. If you do this the oxyphosphate or amalgam filling will wear away, or the porcelain crown will be discolored by the material used. The porcelain will not permit discoloration.

"I do not claim so much originality in it, but still think I have made a step in the right direction in simplifying the methods of crowning, both to patient and operator.

"There is another little advantage in these crowns that will meet many of the wants of the practitioner at a distance. The root can be prepared and a model sent to the depot, and a crown can be returned so nearly what is desired that there is very little trouble in fitting it. With the Logan root there is a doubt whether it will go in its place or not."

In speaking of the wax used, Dr. Genese said he used Parr's, stating that it remains in the box of the tooth and acts as a flux, so that the work is not disturbed in soldering, and the investment does not need to be wet to scald it out. The doctor stated that he did not trust to it entirely, but dusted it with a little calcined borax to prevent the solder from exuding while it is being invested. A patient can ordinarily be dismissed in forty minutes with a perfect crown.

"The investment is Teague's compound, of Aiken, South Carolina, and while on the subject it may be of interest to you to know that this substance is good, not only as an investment for crowns, but for bridgework. In taking impressions of the mouth it can be used exactly the same as plaster. Pure gold can be heated in it, so that it can be understood that zinc or any other metal required can be used. A perfect result is assured. Mix it up with water in the same way as plaster. It can be smoothed, and manipulate it by adding a little water before it is quite set, which will give a perfectly smooth surface. It can be dried out and soldered in ten minutes from the time it is invested. I do not know its composition, but it is made from some fossil remains found in Florida. The Wilmington Manufacturing Company have special machinery for grinding it.

"I soldered that [exhibiting a case] in ten minutes after it was put in the case, a thing impossible with sand or plaster or any other compound.

"I have also found it very satisfactory in bridgework."

The fifth question submitted to the Society was then taken up for discussion, the subject being, "What are the Best Forms for Partial Lower Dentures, and Methods of constructing them?"

The President, Dr. Jack, stated that as he understood it the question was capable of two divisions,—what may be called three-fourths cases and the other full lower dentures. The three-fourths case is confessedly the one most used in dental prosthesis.

Dr. Genese submitted some specimens pertaining to the subject for discussion, and said, "These are all made for practical work; some temporary cases, others duplicates. They embrace most of the forms that will be met with in general practice, from one or two to a complete set. A great deal depends on the margins, the smooth finish of the work, and, above all, absolute freedom from pressure of the muscles of the lower jaw."

The doctor exhibited a case from his own mouth, which he stated he had worn for twenty-three years, and which had been mounted twice, and showed how it had been repaired without a band or fastener of any kind.

"In making partial dentures I dislike to have metal come in contact with the gum. I would trust more to a small wing of rubber to go around the gum to keep them down until the muscles become accustomed to the strange feeling. I take advantage of the leaning forward of the last molars, and get it well under and then drop it down like a bent hook. In complete dentures I use the spiral springs. They are very useful, though I find they are very little known in this country. They are wound with eighteen-carat spring-wire, with a swivel and a small hook and screw between the lower ones to prevent going into the muscles of the jaw, and retain the denture in the mouth exceedingly well for a number of years before breaking."

*Dr. Deane.*—I have had more or less success and a good many failures, but have had more success with gold than with rubber, taking a plaster impression and making the cases very small. As a friend of mine, Mr. Frank Faber, once said, "File your lower case until you think you have spoiled it, and file it again until you think it will fit." I have followed that idea out with about as much success as it is possible to get. Simply fit the ridge of the jaw, and if there is no ridge, still hold to that law, and the articulation being as perfect as it is possible to get it, you will meet with more success than to trust to wide and bulky pieces of rubber or metal.

I think if the pieces passed around are not allowed to lap over the jaws, the fit will be more accurate and they will create less friction on the tooth at the band. I make the band of as near pure gold as I can get, very thin and small. Sometimes I find it necessary to cut it into two or three pieces, and then swedge and solder.

*Dr. Nichol.*—I have prepared these cases, using gold for partial lower dentures where the incisors are in position, and sometimes the first bicuspid, the object being to prevent the plate from pressing to the back part of the mouth. They are used when there is no molar to prevent the plate being driven posteriorly. The plate is swedged in the usual way, the band extending around the ridge just below the incisor tooth, and the whole put in the mouth and filed nicely to fit. An impression is then taken, and the whole invested and soldered in that way. The purpose is to get a band of gold broad enough to prevent cutting into the ridge running around the front of the tooth or the ridge just below the teeth. One of the advantages is to prevent the necessity of using clasps. It is rarely that a bicuspid can be clasped with satisfaction, as it is not adapted for it, and I think this method secures better results than clasping. It avoids the band that Dr. Genese speaks of, and makes a very firm and substantial plate.

*Dr. Bonwill.*—It is not surprising that this query should be made. For nearly forty years I have been looking at the vacancies in the lower jaw from the loss of the second bicuspid and first molar, and often the first bicuspid, leaving the second molar intact, with the cuspid in front, and in many other cases where there is nothing remaining posterior to the six anterior teeth.

The presumption is that the person formulating this question must have felt his own need in practice, but was not conversant with the history of artificial dentistry.

How few dentists have ever undertaken to fill such spaces, and how many failures are made when attempted! I think the question should have been framed, "Have we any means at present for satisfactory restoration of partial spaces in the lower jaw?"

If modern dentistry has a stain upon it, it is that such a question should have to be asked.

Efforts have been made that fully justify me in stating a method that perfectly restores the lower jaw to its normal action. Dentists are certainly either very obtuse or wanting in mechanical manipulation, or prejudiced against any plan or substitute when done by any one else than themselves.

A new method of clasping has lately been shown you that it would be well for every one here to try before he attempts to ask for anything new.

If those present will but recall an article I read before this Society several years ago, and at which time I gave a demonstration in my own mouth, as well as in others, and which for some reason was delayed in publication until a few months ago (see International Dental Journal), I am sure they will find it worthy of some consideration.

To me it has been the addition of a new arm to my practice, and there is no one thing I have ever devised that gives so much satisfaction to my patients and to those dentists who have tried it.

Then, when I am asked such a question as No. 5 of this series of the learned heads of our profession, I feel somewhat like saying to such, Would you know a good thing if you saw it? Why do you, who appreciate the needs, not go to work and do, and not ask to have others exhaust their brains, and then give their efforts no attention?

All I have to say in conclusion is, that if you will read the literature of our profession, and learn to minutely follow details and make yourselves competent in mechanical art and skill, there will not be so many queries made, but we will have effort,—active demonstrations by the dozen coming from every one of you who have the slightest conception of creating a substitute for lost organs in the human jaw.

I would again refer you to the published article in the February number of the INTERNATIONAL DENTAL JOURNAL, and you can see in my own mouth results of years, and a few unfinished cases I have here on exhibition, which, if not enough for the dissatisfied, I cannot offer you any more plans for the present.

*Dr. Deane.*—I think a great deal of the riding backward can be avoided by articulation. If the tooth was so ground as to form an inclined plane, this posterior movement that annoys us a great deal would in a measure be avoided, as I find it quite successful.

